# The Beneficial Role of Natural Endocrine Disruptors: Phytoestrogens in Alzheimer's Disease

**DOI:** 10.1155/2021/3961445

**Published:** 2021-09-03

**Authors:** Anita Domańska, Arkadiusz Orzechowski, Anna Litwiniuk, Małgorzata Kalisz, Wojciech Bik, Agnieszka Baranowska-Bik

**Affiliations:** ^1^Department of Neuroendocrinology, Centre of Postgraduate Medical Education, Marymoncka 99/103, 01-813 Warsaw, Poland; ^2^Department of Physiological Sciences, Institute of Veterinary Medicine, Warsaw University of Life Sciences-SGGW, Nowoursynowska 166, 02-787 Warsaw, Poland; ^3^Department of Endocrinology, Centre of Postgraduate Medical Education, Cegłowska 80, 01-809 Warsaw, Poland

## Abstract

Alzheimer's disease (AD) is the most common form of dementia with a growing incidence rate primarily among the elderly. It is a neurodegenerative, progressive disorder leading to significant cognitive loss. Despite numerous pieces of research, no cure for halting the disease has been discovered yet. Phytoestrogens are nonestradiol compounds classified as one of the endocrine-disrupting chemicals (EDCs), meaning that they can potentially disrupt hormonal balance and result in developmental and reproductive abnormalities. Importantly, phytoestrogens are structurally, chemically, and functionally akin to estrogens, which undoubtedly has the potential to be detrimental to the organism. What is intriguing, although classified as EDCs, phytoestrogens seem to have a beneficial influence on Alzheimer's disease symptoms and neuropathologies. They have been observed to act as antioxidants, improve visual-spatial memory, lower amyloid-beta production, and increase the growth, survival, and plasticity of brain cells. This review article is aimed at contributing to the collective understanding of the role of phytoestrogens in the prevention and treatment of Alzheimer's disease. Importantly, it underlines the fact that despite being EDCs, phytoestrogens and their use can be beneficial in the prevention of Alzheimer's disease.

## 1. Alzheimer's Disease

### 1.1. Introduction

Nowadays, dementia is estimated to affect over 45 million people worldwide and is believed to reach up to 115 million by 2050 [1]. Alzheimer's disease (AD) is a complex, neurodegenerative disorder of the central nervous system (CNS), which accounts for up to 70% of dementias [2]. Dementia can also be caused by other factors, such as brain injuries, vascular disorders, and numerous other diseases, e.g., Parkinson's disease (PD), Huntington's disease (HD), or Creutzfeldt-Jakob disease (CJD).

Alzheimer's disease was first identified more than a century ago by Alois Alzheimer, a German psychiatrist. In 1906, he described a case of dementia observed in a 50-year-old woman. He continued to track the progress of her disease until 1906 when she died [3]. He found at autopsy of her brain characteristic pathological microscopic changes, known today as visible gliosis around numerous senile plaques (SP) and abundant neurofibrillary tangles (NFT) [4]. Currently, about 35 million patients in the world are suffering from Alzheimer's disease.

The etiology and pathogenesis of Alzheimer's disease are complex and despite the efforts of researchers, the mechanisms are still not transparent. Much is yet to be discovered when it comes to precise biological processes. The major challenges include reasons why the disease progresses faster in some patients and slower in others, and finally, how to prevent, stop, or at least slow down the progression of AD [5]. The majority of AD patients (>95%) have sporadic onset, and less than 5% of the cases are related to dominant gene mutations, including APP, PS1, or PS2 genes [3]. Notably, AD is a disease that begins much earlier before its symptoms arise, and according to Alzheimer's Association [5], it can be even up to 20 years earlier. Thus, early detection seems to be crucial for intervention in coping with the disease.

### 1.2. Symptoms and Stages of Alzheimer's Disease

Alzheimer's disease causes both cognitive and noncognitive symptoms and signs. Importantly, the symptoms and progress of AD vary among individuals. This heterogeneity is particularly challenging to patients, their families, and clinicians, considering the difficulty in forecast and recognition functional impairment and other upcoming symptoms [6]. Importantly, AD is characterized by neuronal degeneration in selective brain regions involved in cognition (hippocampus, entorhinal, and frontal cortices) and emotional behaviors (amygdala, prefrontal cortex, and hypothalamus). Current research identifies three stages of AD: preclinical Alzheimer's disease, mild cognitive impairment (MCI) due to Alzheimer's disease, and dementia due to Alzheimer's disease. Symptoms and signs of the disease are present in the last two stages, but importantly with a varying degree [7].

Preclinical AD is a stage in which patients have measurable and significant structural changes in the brain, composition of cerebrospinal fluid (CSF), and presence of blood biomarkers. On the other hand, symptoms such as memory loss are not developed at this point. Importantly, not all individuals with raised Alzheimer's biomarkers progress to develop dementia or mild cognitive decline, but most of them do [8]. Because of no perceptible symptoms, this stage of the disease is notably hard to detect.

MCI due to AD is a stage in which biomarkers such as elevated levels of beta-amyloid protein or neurofilament light chain (NFL) in CSF are raised. Moreover, an individual in this stage of the disease shows cognitive decline much greater than the one expected for her/his age. Importantly, the decline does not always impede patients' everyday activities.

Dementia due to AD is a stage in which prominent thinking, memory, and behavioral impairment affect an individual's daily life. Additionally, evidence of AD-related brain changes is also observed. Patients suffering from the mild stage of Alzheimer's dementia are frequently able to function autonomously in many areas, such as participating in their favorite activities, driving, and working. However, they should be assisted with some tasks to maximize their safety. In the next stage, the moderate stage of Alzheimer's dementia, individuals may find it hard to communicate and conduct their daily tasks, such as taking a shower, dressing up, or brushing their teeth. In this stage, visible changes in their behavior and personality are observed. For instance, they may get irritated easily, become anxious, fearful, or overly suspicious. The last stage of AD is the severe stage of Alzheimer's dementia. Patients suffering from this stage of the disease need help in every area of their lives and are likely to require around-the-clock care. Unambiguous AD diagnosis is classically made postmortem, once neuropathology has confirmed the specific senile plaque and fibrillary tangle deposition in an individual with clinical diagnostic symptoms observed during life.

### 1.3. Hypotheses of the Onset

There are numerous hypotheses when and how it comes to the induction and the beginning of AD. The ones which are considered to be crucial are the inflammation hypothesis, cholinergic hypothesis, tau hypothesis, amyloid *β* (A*β*) cascade hypothesis, and oxidative stress hypothesis.

#### 1.3.1. Inflammation Hypothesis

The inflammation hypothesis suggests that reactive gliosis with coexisting neuroinflammation should be considered crucial in AD pathology. The theory assumes that reactive microglia and astrocytes which surround amyloid plaques secrete numerous proinflammatory cytokines. Thus, they are regarded as an early, prime mover in AD advancement [9].

#### 1.3.2. Cholinergic Hypothesis

In turn, the cholinergic hypothesis is drawn from observations of noticeable cholinergic neuronal cell loss in AD postmortem brains [10]. Besides, neuroscientists confirm the significance of cholinergic neurotransmission in cognitive function, especially in attention and memory encoding [11]. Thus, this hypothesis procured the successful approval of cholinesterase inhibitors in clinical practice [12].

#### 1.3.3. Tau Hypothesis

The tau hypothesis is related to a microtubule-associated protein, tau, which works as a reinforcement for a cytoskeleton in an axon. Under pathological conditions, such as Alzheimer's disease, tau proteins tend to aggregate forming intracellular NFT, thus weakening the cytoskeleton. As a result, not only the structure of the cell is deformed but also the intracellular transport of neurotransmitters encapsulated in vesicles is severely impaired. The aforementioned processes inevitably lead to neurodegeneration [13].

#### 1.3.4. Amyloid *β* Hypothesis

Another important hypothesis concerns the A*β* protein as the main trigger of AD development. Amyloid-beta is produced by endoproteolysis of amyloid precursor protein (APP), encoded by the APP gene. Depending on enzymes that take part in the process, the processing of APP can be divided into two pathways, the nonamyloidogenic pathway and the amyloidogenic pathway [14]. The first pathway, also called the *α* pathway, is conducted by two main enzymes *α*-secretase and *γ*-secretase. As a result, soluble APP (sAPP) is produced, which is not harmful to the organism. The second pathway is called the *β* pathway. In this process, APP is hydrolyzed by *β*-secretase (BACE1) and then by *γ*-secretase, and in consequence, insoluble and toxic A*β* is created. Under normal conditions, A*β* protein is formatted in a very small quantity, since APP cleavage is mainly based on the *α* pathway. Importantly, a limited amount of APP is processed via the second pathway but the A*β* form is efficiently eliminated by the immune system. In unusual conditions, i.e., some mutations of *APP* gene, such as the Lys670Asn/Met671Leu (*Swedish mutation*), APP is prone to be processed by the *β* pathway, resulting in an excessive accumulation of insoluble A*β* and eventually the development of senile plaques [15]. Importantly, A*β* has been the primary target for disease-modifying AD therapies for decades, but so far, A*β*-focused approaches have produced disappointing results in clinical experiments.

#### 1.3.5. Oxidative Stress Hypothesis

Undoubtedly, oxidative stress plays an important role in the pathogenesis of AD. The brain exploits more oxygen than any other organ, and mitochondrial respiration is essential for neurons. Nevertheless, the high demand for oxygen increases the threat of reactive oxygen species (ROS) generation. Numerous researches support the concept that oxidative stress and nitrosative stress have a causative role in the pathogenesis of AD leading to the damage of fundamental cellular elements such as nucleic acids, lipids, and proteins [16]. Additionally, elevated levels of A*β* have been reported to be associated with increased concentrations of oxidation products formed from the aforementioned substances. By contrast, brain regions with low A*β* levels (e.g., cerebellum) did not show any increase in oxidative stress markers [17]. Oxidative stress (OS) refers to a circumstance in which ROS production overwhelms the cellular antioxidant defense systems that consist of antioxidant enzymes, such as superoxide dismutase (SOD), glutathione peroxidase (GPx), catalase, glutaredoxins, and thioredoxins, and also of nonenzymatic antioxidant factors [18]. Importantly, reduction or loss of function of the antioxidant enzymes, as denoted by decreased specific activity, has been reported in AD [19].

#### 1.3.6. Mitochondria Hypothesis

When discussing the impact of OS on AD, it is worth mentioning the role of mitochondria and their dysfunction, through the production of ROS, as an important factor involved in the pathogenesis of AD. Growing evidence, such as documentation of disease staging generated by Alzheimer's Disease Neuroimaging Initiative, supports the notion that AD is not a linear downstream consequence of A*β* or SP deposition alone, but rather should be considered as a multifactorial disease [20, 21]. Some researchers insist that mitochondrial dysfunction is a dominant insult driving the most common, sporadic late-onset AD pathophysiology and refer to this pathology as a “mitochondrial cascade hypothesis” [22].

## 2. Alzheimer's-Like Diseases

Numerous important observations regarding AD and other related dementias have been made in animal studies. Although none of the existing models entirely exhibits the complete spectrum of this insidious human disease, critical aspects of AD pathology and disease process can be effortlessly outlined. Such diseases in animals are oftentimes called Alzheimer's-like diseases (ALD). Interestingly, there is only one group of animals, dogs, which has their own ALD named, and it is called canine cognitive dysfunction (CCD). What is worth highlighting, the vast majority of the transgenic animal models of ALD represent the familial form of Alzheimer's disease. Moreover, there are only a few studies regarding the sporadic form of AD in living animals other than companion animals. The reason for this is surprisingly simple as wild animals suffering from ALD are not able to be studied as neatly as domestic animals are.

Interestingly, many domestic animals exhibit several behavioral changes in their elderly years, e.g., they tend to display spatial disorientation, change relationships with their owners, change their day-night pattern, lose cognition, or simply exhibit inappropriate vocalization [14]. Importantly, except for behavioral symptoms, there are also numerous neuropathological manifestations found in animals that are similar or the same as in AD including A*β* oligomers, senile plaques, neuronal degradation and loss, AD biomarkers found in CSF, vascular amyloid, dysfunction in neurotransmitter systems, decreased neurogenesis, increased oxidative stress, and oxidative damage [23–27].

## 3. Endocrine Disruptors

Notably, scientists presume that AD is mostly caused by a combination of genetic, lifestyle, and environmental factors that affect the brain over time. Many studies point to environmental chemicals as an important component of neurological diseases' onset [28] Synthetic chemicals have become an inseparable part of people's lives, and some of these chemicals have been identified as endocrine disruptor chemicals (EDCs) or endocrine disruptors (EDs).

Endocrine disruptors are exogenous substances, mixtures of chemicals, or nonchemical exogenous factors that interfere with the human endocrine system, leading to adverse effects on hormonally controlled functions [29, 30]. Even though it is facile to automatically assume that EDCs are synthetic substances only, in fact, they are heterogeneous and vary from synthetic to natural chemicals. The best-known synthetic EDCs are polychlorinated biphenyls (PCB), plasticizers, pesticides, fungicides, and pharmaceutical agents. The most popular natural EDCs are phytoestrogens, predominantly found in food and drinks. There are also additional examples of natural EDCs, such as nicotine or, surprisingly, light [31]. Importantly, all of the aforementioned chemicals are widespread in the environment. EDCs interfere with hormone activities by mimicking hormones, promoting responses at improper times, or by halting hormone action, thus leading to alterations in the hormonal and homeostatic systems, and interfering with the ability of the body for communicating and responding to the environmental stimuli. EDCs tend to have a low binding affinity for hormone receptors, and their ability to activate or block hormone receptors may vary. Although it is generally difficult to define a *negative effect*, some researchers consider any biological response to an endocrine disruptor to be an adverse event [32].

Endocrine disruptors can be found in food, consumer products, water, soil, wildlife, and in people who were exposed to EDs through ingestion, inhalation, dermal contact, or injection. Endocrine disruptors can be divided into two large groups: chemicals and nonchemical exogenous factors. Subsequently, chemical EDs can be categorized into three primary groups: pesticides (e.g., glyphosate, dichlorodiphenyltrichloroethane (DDT)), chemicals in consumer products (e.g., parabens and heavy metals), and food contact materials (e.g., bisphenol A (BPA) and phthalates) [33]. When it comes to nonchemical EDCs, a good example could be light [34].

Detrimental properties of EDs are well known to scientists, and importantly, there is a growing awareness regarding them in society. These substances are called *disruptors* not without a reason: exposure to EDs leads to increased incidence and prevalence of cancer; they are associated with the development of learning disabilities, deformations, impairing sexual development; and they can be highly teratogenic [35, 36]. Notably, endocrine disruptors have been linked to numerous diseases, e.g., attention-deficit hyperactivity disorder (ADHD), PD, diabetes mellitus, cardiovascular diseases, obesity, early puberty, infertility and other reproductive disorders, children and adult cancers, and AD [37]. Curiously, EDCs may lead to alterations in microbiota residing in the gut, which in turn may lead to neurobehavioral disorders like autism spectrum disorders [38].

A critical group of endocrine disruptors seems to be the dietary endocrine disruptors. Undeniably, food is essential for human life, and it plays a crucial role in determining the health and well-being of the consumer. Since food is the source of energy for humans, it is also one of the most significant sources of endocrine disruptors in our organisms. Importantly, dietary endocrine disruptors are not originated from food exclusively. Surely, there are numerous EDCs of animal or plant sources just like phytoestrogens, but it is worth mentioning that endocrine disruptors found in food are also pesticides (e.g., endosulfan or carbaryl), metals (e.g., aluminum or antimony), or plasticizers (e.g., bisphenol A or phthalates). When the term endocrine disruptors was used for the first time, scientists used it mainly to discuss the adverse effect of a certain substance on the endocrine system. Oral intake of EDCs was described to result in reduced fertility in men and women; breast, endometrial, and testicular cancer; birth defects of reproductive organs; and changes in the onset of puberty [39]. Nowadays, scientists are aware that the intake of dietary EDCs may lead to numerous diverse side effects. Polychlorinated biphenyls, for example, due to their lipophilic nature, remain global contaminants in the human body. They are proven to have neurotoxic, carcinogenic, immunotoxic, hepatotoxic, nephrotoxic, and cytotoxic effects in numerous experimental models and human studies [40]. Pesticides like carbaryl or endosulfan, in turn, have confirmed influence on developing obesity and metabolic disorders, deteriorating thyroid homeostasis and hypothalamo-pituitary axis, and causing hormone-sensitive cancers [41]. Plasticizers, which eagerly migrate from plastic packaging to food, are known to have serious neurotoxic effects, especially in children and pregnant women [42]. Similarly, the harmful impact has been denoted in the case of metals, which have a potential diabetogenic role and are denoted as EDCs inducing severe testicular damage [43].

Despite their bad fame, endocrine disruptors are increasingly described to have numerous positive side effects, and importantly, many of them are already confirmed by studies. Since AD is still a bit of a riddle for scientists, we decided to shed new light on some well-known EDs and EDCs and their presumed or confirmed roles in AD.

## 4. Phytoestrogens

Phytoestrogens are nonsteroidal, plant-derived compounds that may mimic or interact with estrogen hormones in mammals. They exhibit natural structural similarity to 17*β*-estradiol (E2), which is the most abundant circulating estrogen and the primary female sex hormone at the same time. Thus, phytoestrogens can easily interplay with estrogen receptors (ERs) and mediate estrogenic responses. Curiously, among two currently known forms of estrogen receptors (ER*α* and ER*β*), phytoestrogens have a slightly higher relative binding affinity for ER*β* [44]. Importantly, estrogen receptors' subtype distribution varies across tissues and cell types. Moreover, it changes over the lifespan and is sexually dimorphic [45]. When bound to estrogen receptors, phytoestrogens can commence either transcription or rapid, nongenomic actions via numerous mechanisms and pathways.

Transcription induced by phytoestrogens can be initiated through interactions with the estrogen response element or by binding early immediate genes, such as Jun and Fos [46]. When it comes to the nongenomic actions of phytoestrogens, it is believed that this kind of activity develops at the extracellular surface of the cell membrane, which means that a potential endocrine disruptor does not have to enter the cell in order to be active. The binding of phytoestrogens to ERs activates second messenger pathways, leading to such cellular responses as the rise of intracellular cAMP (cyclic adenosine monophosphate) or calcium levels, or promotion of nitric oxide release contributing to the stimulation of signal transduction pathways crucial, e.g., neuronal signaling or neuronal differentiation [47]. Importantly, a growing number of papers point out that phytoestrogens have an epigenetic activity being able to modify the action of DNA and histone methyltransferases, NAD-dependent histone deacetylases, and other modifiers of chromatin structure [48, 49]. Another molecular mechanism of phytoestrogen's activity is its ability to interfere with certain enzymes required for steroid biosynthesis and/or their degradation. For example, phytoestrogens can inhibit 11*β*-hydroxysteroid dehydrogenase type 1, an enzyme that takes a part in the synthesis of bioactive glucocorticoids from their inactive precursors [50]. Notably, phytoestrogens are also able to affect sex hormone-binding globulin synthesis in the liver and thus affecting sex hormones bioavailability. For example, soya containing a large amount of phytoestrogens is known to lower the risk of some cancers due to heightening sex hormone-binding globulin levels [50, 51].

**Figure 1:**
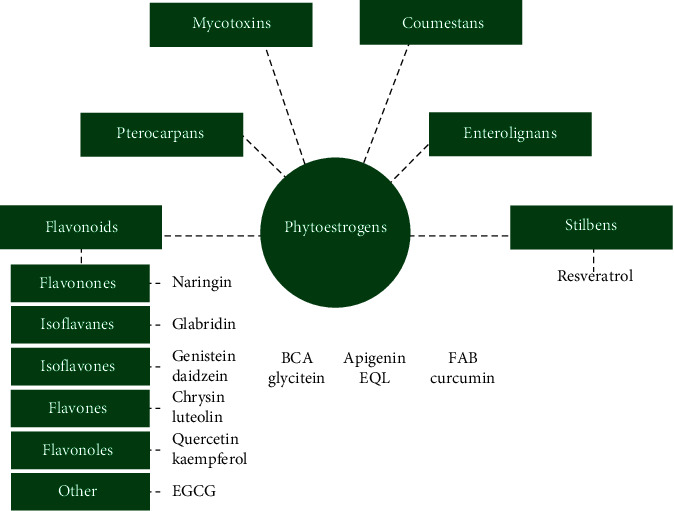
Classification of phytoestrogens with a focus on phytoestrogens described in the article.

Phytoestrogens can be divided into six main classes: flavonoids, pterocarpans, enterolignans, coumestans, mycotoxins, and stilbenes, depending on their chemical structure [52]. Importantly, flavonoids have numerous subclasses, e.g., flavones, flavanones, flavonols, isoflavones, and isoflavanes. The main phytoestrogens derived from the diet are genistein, daidzein, and glycitein, which belong to isoflavones [53]. You can find a graphical classification of phytoestrogens with a focus on phytoestrogens described in the article in Figure 1.

Soy seems to be the richest source of plant estrogens. Interestingly, studies have shown great variability in isoflavone content and composition in soybeans, depending not only on the variety of soy but also on the environmental conditions [54]. Importantly, soybean is prevalently used in the food industry, including milk and meat substitutes, and has become increasingly widespread over the past 20 years. Even the pet-food industry uses heaps of soybeans and soybean-related products in their search. Back in 1998, Setchell suggested that the fertility problems of captive cheetahs could be related to the presence of soy isoflavone phytoestrogens in the standard animal diet [55].

Numerous studies indicate that phytoestrogens can exert adverse effects, especially on reproduction and fertility. Meena et al. performed a study in which pregnant rats received intraperitoneal injections of genistein at a dose level of 2, 20, or 100 mg/kg body weight for 7 days. The results indicated that male rats exposed to phytoestrogens in their mothers' wombs have impaired fertility and altered both spermatogenesis and steroidogenesis [56]. Consistent observations were made in humans in a population-based case-control study by Russo et al., who showed that high consumption of phytoestrogens was associated with a higher occurrence of prostate cancer [57]. Interestingly, even the widely known beneficial effects of soy intake for women with breast cancer are controversial, as *in vitro* studies showed that some phytoestrogens, namely, genistein and daidzein, even in the low concentrations were able to stimulate the proliferation of MCF-7 human estrogen-receptor alpha positive (ER*α*+) breast cancer cells. [58].

### 4.1. Cognitive Function

Importantly, phytoestrogens can also have an impact on the nervous system and behavior. Interestingly, in contrast to the aforementioned systems and organs, the vast majority of the effects of phytoestrogens exerted on cognitive functioning turns out to be advantageous.

#### 4.1.1. Cognitive Function: Human Studies

A meta-analysis of 10 placebo-controlled suitable randomized controlled trials conducted by Cheng et al. in 2015 revealed that supplementation with soy isoflavones indeed improves cognitive function and visual memory in postmenopausal women [59]. What is worth mentioning, researchers underline the importance of geographic features and treatment duration as important factors influencing the effect. A prominent study named SOPHIA was conducted on postmenopausal women [60]. Isoflavones were supplemented to women (110 mg/day), and after 12 weeks of daily supplementation, their cognitive function was assessed. It turned out that the treatment significantly improved performance in the recall of pictures and sustained attention and planning tasks [61].

Sekikawa et al., who proved that equol, a derivative of daidzein, is antiatherogenic and can improve arterial stiffness, stated that equol may help to prevent cognitive impairment or/and dementia [62].

Interestingly, very few studies included males and assessed their reaction to phytoestrogens supplementation. However, these studies provided truly captivating results. A case-control study by File et al. [63] indicated that dietary isoflavone supplementation improved cognitive function in men. Individuals received 100 mg of isoflavones per day for 10 weeks. The isoflavones varied depending on the meal served. Importantly, the diet contained a wide range of soya-containing foods, such as soya milk drinks or puddings, soya flour, or simply soya beans. Young, healthy adults exhibited a significant improvement in both short-term and long-term memory, and additionally, the mental flexibility of studied subjects was improved. Li et al. [64], in turn, pointed out that a diet rich with phytoestrogens affected boys' brains more likely than girls'. In their study, they examined infants fed exclusively with soy formula from the first weeks of their life throughout the first year of life. The study also suggested that a soy-based diet may influence later life brain anatomy and function; however, the changes were modest, and therefore, the results cannot lead to any clinically relevant deficits or abnormal outcomes. Additionally, Thorp et al. [65] showed that oral isoflavone supplementation (capsules with 116 mg of isoflavone equivalents daily: 68 mg of daidzein, 12 mg of genistein, 36 mg of glycitin for six weeks) significantly enhanced spatial memory in men.

#### 4.1.2. Cognitive Function: Animal Studies

Unquestionably, the vast majority of studies regarding the impact of phytoestrogens on the cognitive system have been conducted on animals.

In 2020, Li et al. indicated that genistein has plenty of beneficial effects in diabetes mellitus-induced brain damage [66]. The authors revealed that this phytoestrogen not only improves brain insulin signaling but also increases neurotrophic support and alleviates AD-related pathologies, such as A*β* deposition and the level of hyperphosphorylated tau protein. As a result, cell growth and survival, synaptic plasticity, and cognitive function of such animals are significantly improved. Consistent results were reported by Park et al. [67]. They showed that genistein exerts a protective effect against neurodegeneration in mice [68].

Interestingly, as genistein has intrinsically low oral bioavailability, not only oral delivery of phytoestrogens has been investigated. Rassu et al. suggested that intranasal supplementation of genistein as nanoparticles may be considered to act as a potential preventive system against neurodegenerative disorders [69].

Due to the fact that phytoestrogens are widespread and commonly found in food and drinks, scientists have carried out studies regarding specific fares, dishes, and recipes. A study conducted on mice indicated that a Korean traditional fermented soybean paste named *Doenjang* alleviates neuroinflammation and neurodegeneration [70]. Another research showed that a diet containing high amounts of phytoestrogens not only reduces aggression in mice but also has a strong negative impact on the sociability of the animals [71].

Phytoestrogens, acting as estrogen agonists, can positively affect the synthesis of brain-derived neurotrophic factor (BDNF) and nerve growth factor (NGF). This hypothesis was confirmed back in 1999 by Pan et al., who showed the mRNA levels of BDNF in the frontal cortex were significantly higher in rats receiving soy isoflavones compared to those fed without any phytoestrogens [72]. NGF, in turn, increases the mRNA of choline acetyltransferase and increases its activities, thus promoting the release of acetylcholine. Importantly, NGF has an outcome principally on cholinergic neurons, which are the most prone to neurodegeneration in AD. Moreover, certain studies pointed out that NGF can be crucial in slowing the progression of AD since it impedes cholinergic basal forebrain atrophy. As a result, phytoestrogens, due to their ability to promote NGF and NGF receptor expression, are more and more often believed to be potential preventative treatment against AD [73].

### 4.2. Nervous System

#### 4.2.1. *In Vitro* Neuroprotection

Numerous studies have already indicated that phytoestrogens have neuroprotective effects against varied kinds of damage. This fact has been proven using a wide range of in vitro models.

Phytoestrogen isoflavones, principally biochanin A (BCA), significantly increase the expression of glutamate oxaloacetate transaminase (GOT), an enzyme that can metabolize neurotoxic glutamate, in mouse hippocampal HT4 neural cells [74]. A consistent result was confirmed by Tan et al., who indicated that BCA had strong neuroprotective effects against *β*-amyloid-induced neurotoxicity in PC12 cells [75]. This effect is exerted via a mitochondria-dependent intrinsic apoptotic pathway. Another widely known isoflavone, apigenin, was found to give an antiapoptotic effect in murine HT22 hippocampal neuronal cells as well as in human SH-SY5Y neuroblastoma cells [76, 77]. Additionally, apigenin can reduce glutamate-induced Ca^2+^ signaling in cultured murine cortical neurons [78]. Combined results indicate that this isoflavone possesses strong neuroprotective properties.

Equol (EQL), a metabolite of dietary daidzein (DAI), may mitigate the activation of BV-2 microglia. It also enhanced the neuroprotection of C6 astrocytes and N2a (Neuro2a) neuroblastoma cells [79]. One of the most recent studies regarding *in vitro* effects of EQL showed that this metabolite also had a neuroprotective effect against neurotoxins-induced toxicity in SH-SY5Y human neuroblastoma cells [80].

Quercetin is a well-known plant-derived antioxidant. It is one of the predominant flavonoids found in our daily diet. It has been revealed that quercetin decreased the maturation of APP, which in turn alters A*β* secretion and aggregation [81]. Additionally, quercetin glycosides have confirmed neuroprotective effects in such diseases as AD and PD. Magalingam et al. conducted an experiment, in which PC-12 cells pretreated with isoquercitrin and rutin were later exposed to 6-hydroxydopamine (6-OHDA), a synthetic, neurotoxic compound [82]. Cells exposed to glycosides markedly attenuated the expression of proapoptotic genes, such as *Casp1*, *Casp3*, and *Casp7*, when compared to the control cells.

Consistent research was carried out by Kim et al., in which quercetin and kaempferol demonstrated their neuroprotective effects by downregulating the expression of proapoptotic proteins in human SH-SY5Y cells [83]. Additionally, the aforementioned phytoestrogens significantly increased the viability of cells treated with A*β*.

Importantly, phytoestrogens showed a beneficial impact on cells not only in cellular models of AD. Abbruzzese et al. experimented with a startling outcome: genistein and BCA played dual roles in the regulation of autophagy [84]. Depending on whether autophagy is a neuroprotective and prosurvival mechanism or prodeath mechanism, they acted either as autophagy initiation enhancers or as autophagy initiation inhibitors. The experiment was conducted on cortical neurons of Wistar rats in the model of ischemia that is a crucial process in many neurodegenerative diseases.

Flavonoid agathisflavone (FAB) is a phytoestrogen derived from the plant *Poincianella pyramidalis*. FAB significantly ameliorated neuroinflammation induced by LPS (lipopolysaccharide) and proinflammatory cytokines in cocultures of glia and neurons. This finding undoubtedly indicates that FAB has not only neuroprotective but also anti-inflammatory effects *in vitro*, which, in turn, may be considered as an ancillary for the treatment against numerous neurodegenerative diseases [85].

#### 4.2.2. *In Vivo* Neuroprotection

Neuroprotective effects of phytoestrogens have been proven multiple times in *in vivo* models of AD. Ipriflavone (IPRI) is a nonhormonal isoflavone used in some countries as a treatment and prevention against osteoporosis caused by menopause. In a study by Hafez et al. on a rat model, IPRI turned out to play a great role in AChE inhibition, alleviated oxidative stress, reverted memory impairment, and, importantly, significantly increased the expression of two putative *α*-secretase enzymes: ADAM10 and ADAM17 [86]. Additionally, IPRI increased the expression of pERK1/22 (phosphorylated extracellular signal-regulated kinase 1, 2), a serine-threonine kinase that plays a crucial role in decreasing the BACE expression in the hippocampus. What is promising, IPRI significantly reduced tau and A*β* pathologies.

Apigenin is a flavonoid widely spread among fruits and vegetables, such as parsley, oranges, or onions. It has anti-inflammatory, antioxygenic, antitumorigenic, and antimutagenic effects on numerous cell types [76, 87]. Importantly, apigenin plays also an important role in AD. Scientists have reported that apigenin inhibited oxidative stress in APP/PS1 double transgenic mice. Additionally, apigenin ameliorated AD-associated memory impairment in mice and reduced the spread of A*β* plaques [88].

Quercetin is another phytoestrogen with strong anti-AD potential studied *in vivo*. In a study by Sabogal-Guaqueta et al., quercetin was administrated every 48 hours for three months on aged (21-24 months old), triple-transgenic AD model mice (3xTg-AD). Quercetin decreased not only extracellular *β*-amyloidosis but also microgliosis, astrogliosis, and tauopathy in the hippocampus and amygdala. Additionally, a visible reduction in *β*-amyloid 1-40 and *β*-amyloid 1-42 levels was observed. However, not only histological changes were observed but also quercetin had an enhancing effect on learning spatial memory tasks and improved risk-assessment behavior [89]. A similar conclusion was carried out by Shveta et al., who also confirmed the neuroprotective role of quercetin, but this time, the experiments were conducted on rats [90].

Numerous papers indicated that less-known phytoestrogens have also neuroprotective effects; however, these substances are still not studied sufficiently. A good example of this group could be naringin, a flavonoid found in citrus fruits, which weakens oxidative stress and neuroinflammation *in vivo* [91]. Some other phytoestrogens which have confirmed neuroprotective impact *in vivo* and are not as widely known as aforementioned compounds are luteolin (LUT), commonly found in spices like parsley or thyme [92]; chrysin (CHR), a flavonoid that interacts with TTR protein (transthyretin) and thus TTR can play its role and take a part in A*β* clearance [93; 94]; epigallocatechin gallate (EGCG), which is the major polyphenol component of green tea, has confirmed neuroprotective effect *in vivo* not only in AD but also in amyotrophic lateral sclerosis (ALS), multiple sclerosis (MS), and PD. EGCG, just like CHR, can bind to TTR and at the same time suppress *β*-amyloid fibril formation [93]. Neuroprotective effects *in vivo* were also observed with *γ*-mangostin (*γ*-M), originally isolated from *Garcinia mangostana* tree [94] and glabridin (GLA) isolated from the roots of *Glycyrrhiza glabra* L. [95–97].

Naturally, phytoestrogens have neuroprotective effects not only in AD models but also in a plethora of other neurodegenerative diseases. A study by Ohgomori et al. [98] showed that genistein alleviated the demyelination of mature mice oligodendrocytes induced by cuprizone (CPZ), and thus, phytoestrogens are considered to have therapeutic potential for treating patients with multiple sclerosis or those suffering from mental health disorders. Khanna et al. conducted an experiment on C57BL/6 mice, which were intraperitoneally injected with biochanin A for 4 weeks and afterward subjected to ischemic stroke injury [74]. It turned out that BCA treatment in mice induced GOT expression, attenuated stroke lesion volume, and improved sensorimotor functions. Furthermore, in the rat model of PD, BCA decreased the levels of proinflammatory cytokines such as IL-6, IL-1*β*, and TNF*α* and at the same time inhibited the generation of reactive oxygen species. In this study, male Sprague-Dawley rats were treated with BCA for 21 days [99]. Another study by Wang et al. [99] revealed that BCA attenuated behavioral deficits and dopamine depletion induced by the combined effect of iron and rotenone in Sprague-Dawley rats. Equol was confirmed to have neuroprotective possibilities against neurotoxicity induced by 1-methyl-4-phenylpyridinium (MPP+) using *in vivo* model of PD including *Caenorhabditis elegans* (*C. elegans*). Importantly, EQ prolonged the survival of *C. elegans* exposed to MPP+ from 72 to 108 hrs [80].

### 4.3. Cerebral Ischemia Injury

There is a growing number of data indicating the relationship between brain ischemia and Alzheimer's disease [100]. Besides, several studies revealed epidemiological and neuropathological factors linking ischemic brain neurodegeneration with the genotype and phenotype of Alzheimer's disease. Furthermore, Alzheimer's disease is a risk factor for stroke, and conversely, stroke enhances the risk of AD. Finally, dysregulation of Alzheimer's disease-associated genes including *amyloid protein precursor*, *α-secretase*, *β-secretase*, *presenilin 1*, *presenilin 2*, and *tau protein* occurs in a course of postischemic neurodegeneration [100]. Oxidative stress (OS) and neuroinflammation are crucial mechanisms in the progression of cerebral ischemia injury. Importantly, they both are components in the etiology of neurodegenerative diseases. Thus, maintaining physiological blood flow, halting OS, and limiting neuroinflammation are essential in the prevention of brain injuries [101].

Ever since it was denoted that men have a higher incidence of stroke compared with premenopausal women [102], estrogens are claimed to possess neuroprotective properties. This finding along with the widespread use of estrogens as hormone-replacement therapy (HRT) for amending postmenopausal syndromes caused a large number of reports insisting that estrogens may have neuroprotective effects on brain stroke. However, the results of numerous *in vivo* and *in vitro* studies are ambiguous as some confirm this hypothesis, whereas others claim that estrogens can be simply detrimental for postmenopausal women because of the potential deleterious side effects of hormone replacement therapy, such as increased cancer and stroke risk [103]. Thus, a lot of expectations are held concerning phytoestrogens, which seem to be the best and the safest alternative to estrogens.

Several phytoestrogens have been shown to strengthen endogenous antioxidant defenses. Some of them activate NRF2 (nuclear factor erythroid 2-related factor 2) and its gene targets, which are regulated by ARE (antioxidant response element) DNA sequence. Activation of the NRF-2/ARE pathway provokes the production of antioxidant enzymes, such as heme oxygenase-1 (HO-1) or NAD(P)H dehydrogenase (quinone 1) (NQO1). The examples of phytoestrogens that can activate the pathway in neurons are quercetin, curcumin, resveratrol, sulforaphane, epigallocatechin gallate, piceatannol, or brazilin [104, 105]. Some of the aforementioned phytoestrogens, like quercetin or curcumin, can additionally increase the expression of protein chaperones, involving heat-shock proteins and numerous growth factors such as BDNF, IGF (insulin-like growth factor), or FGF (fibroblast growth factor) [106]. Protein chaperones bind to other proteins, protect them, and mediate the unfolded protein response in the endoplasmic reticulum which ensues in neurons during ischemic stroke. Importantly, heat-shock protein response is a well-known and very important process of defense against ischemic stroke-induced stress conditions [107]. Heat shock proteins take a part in protein folding and protection and removal of aggregated proteins, and they could inhibit apoptotic cell death cascade [108].

## 5. Hormone Replacement Therapy

Some researches indicate that the risk of AD incidence and the severity of the disease differ indispensably between men and women. It is estimated that women suffer from the disease even twice as often as men. Although this discrepancy could be partially explained by differences in mean length of life, other mechanisms cannot be omitted. According to recent findings, a decrease in estrogen levels in the menopause period could be connected with AD-related brain changes [109]. Additionally, premature menopause also increases the risk of AD onset or development [110].

A question could be raised why estrogen depletion is so harmful to cognition processes. From the physiological point of view, estrogens act through a wide network of receptors. Two types of receptors could be distinguished, estrogen receptor alpha (ER-*α*) and estrogen receptor beta (ER-*β*). ER-*α* acts as a neuroprotective element against AD by maintaining intracellular signaling cascades [111]. ER-*β* is a potent regulator of the innate immune response as well as is involved in the regulation of neuronal mitochondrial function [112]. Besides, reduced expression of ER-*α* has been found in hippocampal neurons of AD subjects. Furthermore, lower expression of ER-*β* was related to abnormal mitochondrial function and enhanced OS markers [111] A midlife change in neurohormonal activity in females is called the menopause transition. In this particular phase of women's life, there is a decline in the production and secretion of estrogens, mainly estradiol, from the ovaries. As an effect of the decrease of estrogen concentration, several changes in the brain are observed. Among them, there is a low estrogen-related metabolic dysfunction leading to the hypometabolic state [113].

Since data suggest that perimenopause increases a patient's vulnerability to developing not only AD but also other neurological diseases like PD, it may be a promising way to use hormonal replacement therapy for favorable effects on female cognitive function [114]. The coherent conclusion was also conferred by the group from Mayo Clinic. According to them, women who underwent bilateral or unilateral oophorectomy before the onset of natural menopause had a lifelong increased risk of cognitive impairment and dementia [115, 116].

Numerous research reviews, meta-analyses, and *in vivo* and *in vitro* experiments were conducted to examine the link between estrogen replacement therapy (HRT) and its effect on AD. In general, the results were promising. Imtiaz et al. [117] showed in their cohort study that long-term HRT was associated with a reduced risk of AD. The consistent result was presented by Song et al. in their meta-analysis [114]. These authors confirmed that estrogen replacement therapy decreases the risk of onset and/or development of not only AD but also PD. What is worth mentioning, recent reviews indicate that the positive outcome of HRT use in the case of AD may depend on such factors as the length of hormone administration, duration of treatment, and an individual's risk of developing cancer or/and stroke [118].

Of note, an interesting study was performed by Luoto et al., who assessed the impact of HRT on women's health and lifestyle. It turned out that 28% of HRT users had higher education, lived in the capital city's area, had a significantly higher healthy diet factor score, and were leaner than nonusers of HRT. Importantly, the use of HRT remained a significant determinant of body mass index, waist/hip ratio, and body fat percentage [119]. In addition, a recently published meta-analysis indicated that higher levels of education were connected with false negatives of the MMSE (the MiniMental State Examination), whereas lower levels of education were associated with false positives [120].

However, except for the optimistic results, some findings put in doubt the use of estrogens not only in the prevention of AD but also, if not primary, in hormone replacement therapy. Savolainen-Peltonen et al. [121] in their case-control study indicated that for women who initiated hormone replacement therapy before the age of 60, the use of HRT was associated with a 17% increase in the risk of AD. The percentage was even higher in women who initiated HRT after the age of 60, and it reached up to 38%. Besides, not only an enhanced risk of AD and PD was observed in elderly women treated with estrogens but also increased mortality was indicated [119]. However, it should be highlighted that there is a critical period of effective estrogen treatment following menopause. The duration of this advantageous time depends on the depletion of estradiol receptors, the switch to a ketogenic metabolism by neuronal mitochondria, and a decrease of acetylcholine accompanying estradiol deficiency [122].

Interestingly, the side effects of HRT did not affect the nervous system only. For years, it was almost a dogma that any cardiovascular disease was prevented in women undergoing HRT. However, the Women's Health Initiative (WHI) trial of conjugated equine estrogens (CEE) and medroxyprogesterone acetate (MPA) showed in 2002 that HRT resulted in a significantly increased incidence of stroke and venous thromboembolism [123–125]. Because of the WHI results and other side effects of HRT, a lot of effort nowadays is put into finding a less harmful, but still efficient alternative to estrogens. Thus, phytoestrogens seem to be a very promising alternative. Importantly, phytoestrogens have an exceptional ability to act as estrogen agonists without any currently known detrimental effects. They could be the perfect solution and reconcilement between the positive impact of estrogens on AD and their harmful effects on health. The enthusiasm about the use of phytoestrogens emerged from epidemiologic studies indicating that PEs lower the risk of breast cancer and osteoporosis and mitigate the symptoms of menopause in women from countries with high phytoestrogens consumption [128, 129]. Additionally, some of the phytoestrogens own antioxidant properties *in vitro* through hydrogen/electron donation *via* hydroxyl groups; hence, they act as free radical scavengers and can suppress the progression of coronary heart disease and some types of cancer [126]. PEs are also able to upregulate the expression of manganese superoxide dismutase (MnSOD) and catalase, which enhances their antioxidant effect [125, 127]. In Table 1, you can find the summary of processes and organs affected by phytoestrogens and articles cited in the article regarding them.

There are two mechanisms by which phytoestrogens exert their impact: estrogen-mediated and nonestrogen-mediated. The nonestrogen-mediated mechanisms are inclusive of any reaction in which phytoestrogen plays a unique role. This means that phytoestrogens do not compete with estrogens but simply do their “own job.” They protect neurons against OS and prevent the damage it causes [128]. The estrogen-mediated mechanisms, in turn, are inclusive of all reactions in which estrogen is a factor [73]. Substantially, in these mechanisms, phytoestrogens substitute estrogens when they are in low concentrations by simply binding to estrogen receptors. Examples of such mechanisms are reduction of tau protein phosphorylation, reduction of A*β*, promotion of Ca^+^ outflow, or enhancing acetylcholine release [129].

### 5.1. Nonestrogen-Mediated Mechanisms

#### 5.1.1. Antioxidant Properties and Effects on Neurons

Certain amyloid-beta peptides are known for having toxic properties. Phytoestrogens, in turn, often act as their functional antagonists [134].

A*β* is among the factors that stimulate lipid peroxidation in the neuronal cell membrane, the process that leads to the production of reactive oxygen species and respiratory burst. Subsequently, membrane proteins can be impaired and the homeostasis of ion distribution could be broken. As a consequence, the neuronal membrane depolarizes resulting in Ca^2+^ inflow via the *N-methyl-D-aspartate* (NMDA) receptor channels. As a result of such phenomena, the damage of lipids and DNA aggravates even more, eventually leading to neuronal death.

Auspiciously, numerous researches indicate that estradiol (and in turn phytoestrogens) is a natural antioxidant for membrane lipid peroxidation, and it mitigates A*β* toxicity against neurons [135–138]. Importantly, phytoestrogens significantly improve cerebral blood circulation and, therefore, increase oxygen and nutrient supply to the brain cells [139].

### 5.2. Estrogen-Mediated Mechanisms

#### 5.2.1. Decrease in A*β* Production

What should be underlined once more is that not all APPs are detrimental to health. A large number of APPs is, in fact, advantageous. They are essential for synapses' formation, neuronal plasticity, or iron export. APPs may sustain cognitive functioning in patients with AD and ALD [73]. However, some APPs are precursors for neurotoxic A*β*.

Estrogens are known to regulate the metabolism of APP. They can promote the release of APPs in cortical neurons via the protein kinase C pathway. Phytoestrogens, in turn, may act as an alternative if the concentration of estrogens is not high enough, resulting in the same outcome. Regarding their ability to stimulate the excessive formation of APP in elderly individuals, phytoestrogens change the ratio between APP and A*β* in favor of APPs. Eventually, the A*β* production is reduced and serves antagonistic to the progression of AD [140, 141].

#### 5.2.2. Decrease of Tau Protein Phosphorylation

Tau phosphorylation is one of the hallmarks of AD and ALDs. Once tau protein gets phosphorylated and converts to NFT, it destabilizes the structure of cell's cytoskeleton and impairs the functioning of a neuron and eventually leads to its death.

Estrogens are known for their anti-tau-phosphorylation features. So are phytoestrogens, exerting their effects by enhancing dephosphorylation in the proline-rich region of the tau molecule [142, 143]. It has been observed that phytoestrogens can significantly inhibit tau phosphorylation and fibrillation and the associated cytotoxicity [144]. Additionally, according to findings of numerous *in vivo* and *in vitro* studies, inhibition of tau phosphorylation prevents endoplasmic reticulum stress-mediated neurodegeneration [145–147].

#### 5.2.3. Enhancing Acetylcholine Release

Since phytoestrogens act as estrogens' agonists, they similarly can affect processes that involve estrogens, such as promoting axon pruning, synaptic growth, or the expression of numerous factors, e.g., NGF. NGF plays an important role in the maintenance of cholinergic neuron integrity and function, both during development and adulthood. Importantly, NGF induces the expression and activity of acetylcholine esterase (AChE), a key enzyme to degrade acetylcholine in cholinergic synapses [148]. As a result, the release of acetylcholine is promoted. It is important to note that cholinergic neurons are the target neurons for deterioration in AD and ALDs.

Many pieces of research showed that NGF plays an important role in delaying or even stopping the progression of AD as it fights cholinergic basal forebrain atrophy [149, 150]. Thus, more and more reports nowadays suggest that NGF, NGF receptor (NGF-R), and phytoestrogens promoting expression of NGF and NGF-R should be concerned as a potential preventative agent or, hopefully, even as a promising treatment against AD [151, 152].

#### 5.2.4. Promotion of Ca^2+^ Outflow

Phytoestrogens like estrogens can inhibit the elevation of intracellular Ca^2+^ concentration and reverse the disequilibrium of calcium homeostasis often caused by A*β*. They achieve these results through the release of the intracellular Ca^2+^ via nongenomic signaling events, which are not influenced by extracellular Ca^2+^ concentration [73, 153, 154].

Appropriate calcium homeostasis is crucial among individuals suffering from AD. Curiously, it is prevalent among this group of patients since the imbalance of Ca^2+^ is caused by A*β* peptides. When the homeostasis is unbalanced, numerous adverse effects can occur: calcium channels may be activated, there may be changes in the release of neurotransmitters, the neurilemma can be altered, and eventually, the apoptotic signaling cascade can be induced [71, 155].

**Figure 2:**
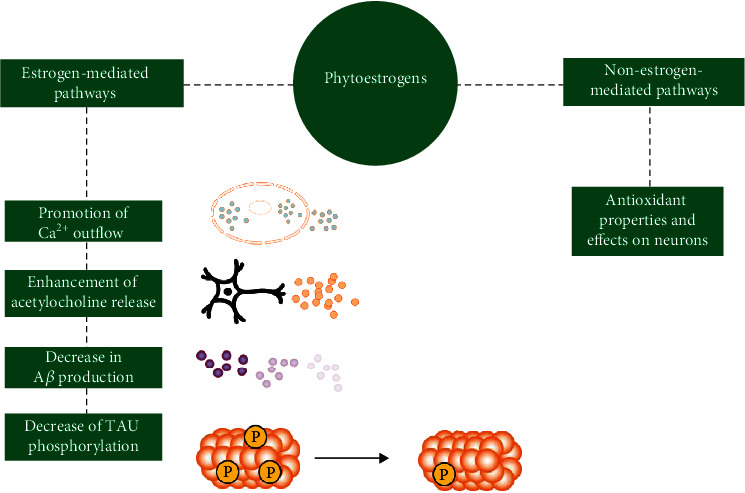
Estrogen- and nonestrogen-mediated mechanisms exerted by phytoestrogens on neurons.

Nowadays, phytoestrogens are widely studied in terms of their calcium-equilibrating properties, and the results are truly satisfactory. A lot of attention in these terms is attracted by genistein and other flavonoids, suggesting the importance of adequate nutrition for humans and animals [155, 156]. You can see estrogen- and nonestrogen-mediated mechanisms exerted by phytoestrogens on neurons in Figure 2.

## 6. Conclusions

AD is a progressive, irreversible neurodegenerative disease affecting a growing cohort of patients. Alzheimer's-like diseases (ALDs), in turn, are group of disorders, closely related to AD, from which our friends and life companions, animals, suffer frequently [14]. Even though numerous researches, regarding the diseases, are published daily and the knowledge about AD and ALDs is constantly growing, there is still no discovered nor invented drug, which would reverse or ultimately inhibit the development of the disease. Moreover, the medications that are already in use provide symptomatic treatment only. Besides, many of them tend to lower their effectiveness over time [157]. Furthermore, drugs that are broadly used nowadays, like rivastigmine, donepezil, or memantine, have multiple side effects impeding the everyday life and functioning of patients. Therefore, undoubtedly, a new approach should be concerned in terms of fighting against AD and ALDs.

Although phytoestrogens are commonly known as endocrine disruptors and by definition could have a negative impact on the endocrine system, they also have an enormous potential to influence cognition and brain functioning beneficially. Numerous *in vitro* and *in vivo* studies confirm that they do have neuroprotective and antioxidant effects and indeed mitigate the progression of AD in humans as well as ALDs in animals. Together with their widespread availability, multifariousness, and their positive impact on other disorders and ailments, phytoestrogens appear to be a promising medicament in the proximate future. Needless to say, more research is needed to provide indisputable confirmation of phytoestrogens' role not only in AD and ALDs but also in other aspects of well-being.

After all, an ancient proverb says “*The enemy of my enemy is my friend*”. Hence, perhaps we should stop considering phytoestrogens as disruptors only, and focus on “our” evident, mutual opponents, such as Alzheimer's and Alzheimer's-like diseases.

## Figures and Tables

**Table 1:** Summary of systems and processes influenced by phytoestrogens mentioned in the publication (with responding references).

Process/organ influenced by phytoestrogens	Specification	Reference number
Cognitive function	Human studies	[59–65]
	Animal studies	[66, 67, 68, 69, 70, 71, 72, 73]

Nervous system	*In vivo*	[74, 76, 80, 86–99, 130, 131]
	*In vitro*	[74–85]

Cerebral ischemia injury	—	[100–108]

Hormone replacement therapy	—	[73, 109–129, 132, 133]

## Data Availability

Please contact with the main author of the work by email: adomanska@cmkp.edu.pl.

## Abbreviations

6-OHDA: 6-hydroxydopamine
AChE: Acetylcholinesterase
AD: Alzheimer's disease
ALD: Alzheimer's-like diseases
ALS: Amyotrophic lateral sclerosis
APP: Amyloid precursor protein
ARE: Antioxidant response element
A*β*: Amyloid *β*
BACE1: *β*-Secretase
BCA: Biochanin A
BDNF: Brain-derived neurotrophic factor
BPA: Bisphenol A
cAMP: Cyclic adenosine monophosphate
CCD: Canine cognitive dysfunction
CEE: Conjugated equine estrogens
CHR: Chrysin
CJD: Creutzfeldt-Jakob disease
CNS: Central nervous system
CPZ: Cuprizone
CSF: Cerebrospinal fluid
DAI: Daidzein
DDT: Dichlorodiphenyltrichloroethane
E2: 17*β*-estradiol
ED: Endocrine disruptor
EDC: Endocrine disruptor chemical
EGCG: Epigallocatechin gallate
EQL: Equol
ER: Estrogen receptors
ER-*α*: Estrogen receptor alpha
ER-*β*: Estrogen receptor beta
FAB: Flavonoid agathisflavone
FGF: Fibroblast growth factor
GLA: Glabridin
GOT: Glutamate oxaloacetate transaminase
GPx: Glutathione peroxidase
HD: Huntington's disease
HO-1: Heme oxygenase-1
HRT: Hormone-replacement therapy
IGF: Insulin-like growth factor
IPRI: Ipriflavone
LPS: Lipopolysaccharide
LUT: Luteolin
MCI: Mild cognitive impairment
MnSOD: Manganese superoxide dismutase
MPA: Medroxyprogesterone acetate
MPP+: 1-methyl-4-phenylpyridinium
MS: Multiple sclerosis
NFL: Neurofilament light chain
NFT: Neurofibrillary tangles
NGF: Nerve growth factor
NGF-R: NGF receptor
NMDA: N-methyl-D-aspartate
NQO1: NAD(P)H dehydrogenase
NRF2: Nuclear factor erythroid 2-related factor 2
OS: Oxidative stress
PCB: Polychlorinated biphenyls
PD: Parkinson's disease
ROS: Reactive oxygen species
sAPP: Soluble amyloid precursor protein
SOD: Superoxide dismutase
SP: Senile plaques
TPO: Thyroid peroxidase
TTR: Transthyretin
WHI: Women's Health Initiative
*γ*-M: *γ*-Mangostin.
